# Sevoflurane as bridge therapy for plasma exchange and Anakinra in febrile infection–related epilepsy syndrome

**DOI:** 10.1002/epi4.12545

**Published:** 2021-10-20

**Authors:** Manuela L’Erario, Rosa Maria Roperto, Anna Rosati

**Affiliations:** ^1^ Intensive Care Unit Meyer Children’s Hospital Florence Italy; ^2^ Nephrology Department Meyer Children’s Hospital University of Florence Florence Italy; ^3^ Paediatric Neurology Unit Meyer Children’s Hospital University of Florence Florence Italy

**Keywords:** children, FIRES, NORSE, plasma exchange, refractory status epilepticus, sevoflurane

## Abstract

Febrile infection–related epilepsy syndrome (FIRES) is a devastating immune inflammatory–mediated epileptic encephalopathy. Herein, we discuss a previously healthy 8‐year‐old boy with FIRES in whom high dosages of conventional and nonconventional anesthetics were ineffective in treating SE, as were ketogenic diet, intravenous corticosteroids, and immunoglobulins. After 29 days of prolonged SRSE, the patient was successfully treated with sevoflurane paired with plasma exchange, for a total of five days, thus obtaining a stable EEG suppression burst pattern with no adverse events. Anakinra at the dosage of 100 mg b.i.d. was started seven days after sevoflurane and plasma exchange had been discontinued and was effective in ensuring non‐recurrence of SE. Sevoflurane as bridge therapy for immunosuppressive treatment could be considered an early, safe, and effective option in treating convulsive SE in which an autoimmune‐inflammatory etiology can reasonably be hypothesized.

**Key Points**

- Sevoflurane such as isoflurane should also be considered in treating refractory convulsive status epilepticus in children.
- Sevoflurane as bridge therapy for plasma exchange and anakinra in treating convulsive status epilepticus of presumed or ascertained autoimmune‐inflammatory etiology.
- Lower concentration, a shorter anesthesia induction time and benzodiazepine co‐administration could reduce sevoflurane toxicity and enhance its efficacy.

## 1 INTRODUCTION

Febrile infection–related epilepsy syndrome (FIRES), which has now been classified as a subset of new‐onset refractory status epilepticus (NORSE), is a devastating epileptic encephalopathy that typically begins with a mild nonspecific febrile illness in an otherwise healthy individual.^1^ Twenty‐four hours to two weeks after a febrile episode, seizures appear and evolve into a prolonged super‐refractory status epilepticus (SRSE) which may continue for months.^2^

Bilateral and multifocal seizure onset with autonomic features and a strong tendency to evolve into bilateral tonic‐clonic seizures is commonly observed. Specific EEG patterns have been recognized in patients with FIRES.^3^ Common sequels are drug‐resistant epilepsy, cognitive impairment, and psychosis. The etiology is unknown. An underlying inflammatory or immune mechanism, however, has been hypothesized based on seizure appearance after a febrile episode and the evidence of intrathecal overproduction of proinflammatory cytokines and chemokines.^4^ Based on its hypothesized autoimmune etiology, patients with suspected FIRES are generally treated with intravenous corticosteroids, immunoglobulins, plasma exchange, and immunotherapy.^5^, ^6^, ^7^, ^8^ Several treatments, including ketogenic diet, hypothermia, vagus nerve stimulation, and cannabidiol have been reported and proposed for the treatment of FIRES but no records exist on the use and efficacy of inhaled anesthetics.^5^, ^6^, ^9^ Increasing evidence indicates anakinra, an IL‐1 receptor antagonist, as a safe and efficacious therapy in FIRES.^10^ Anakinra efficacy has also been reported in improving recovery and long‐term outcomes; its early administration has therefore recently been recommended in children with suspected FIRES.^10^ The efficacy of inhalational anesthetics has been reported in the treatment of RCSE.^11^ Isoflurane is the only inhaled anesthetic recommended and is preferred over desflurane, xenon, and sevoflurane because of its higher rate of tolerability and hypothetical lower toxicity.^11^ Sevoflurane is a halogenated inhaled anesthetic with rapid onset and short‐lasting activity, with proven efficacy and safety for both the induction and maintenance of general anesthesia.^12^ In comparison with older inhalational agents such as isoflurane or halothane, the most important property of sevoflurane is its low solubility in the blood. This results in a more rapid uptake and induction than the 'older' inhalational agents, improved control of depth of anesthesia, and faster elimination and recovery.^12^

Here we report a case of an 8‐year‐old boy with FIRES in whom inhalation anesthesia with sevoflurane as bridge therapy for immunosuppressive treatment with plasma exchange and anakinra was effective and safe in controlling a prolonged SRSE.

## 2 CASE REPORT

An otherwise healthy 8‐year‐old boy with a recent febrile status was transferred to the pediatric iIntensive‐care unit (PICU) of Meyer Children's Hospital from a second‐level hospital due to a coma status and recurrent focal motor seizures. Blood and cerebrospinal fluid exams were unremarkable, and a clear infective etiology was not identified. Previously the patient had been unsuccessfully treated with endorectal diazepam and intravenous benzodiazepines, phenytoin and phenobarbital. Upon admission to our hospital, the patient was intubated and underwent continuous video‐electroencephalography (EEG) monitoring which recorded right and left focal motor seizures and secondarily generalized seizures fulfilling the criteria for refractory SE.^1^, ^2^

**FIGURE 1:**
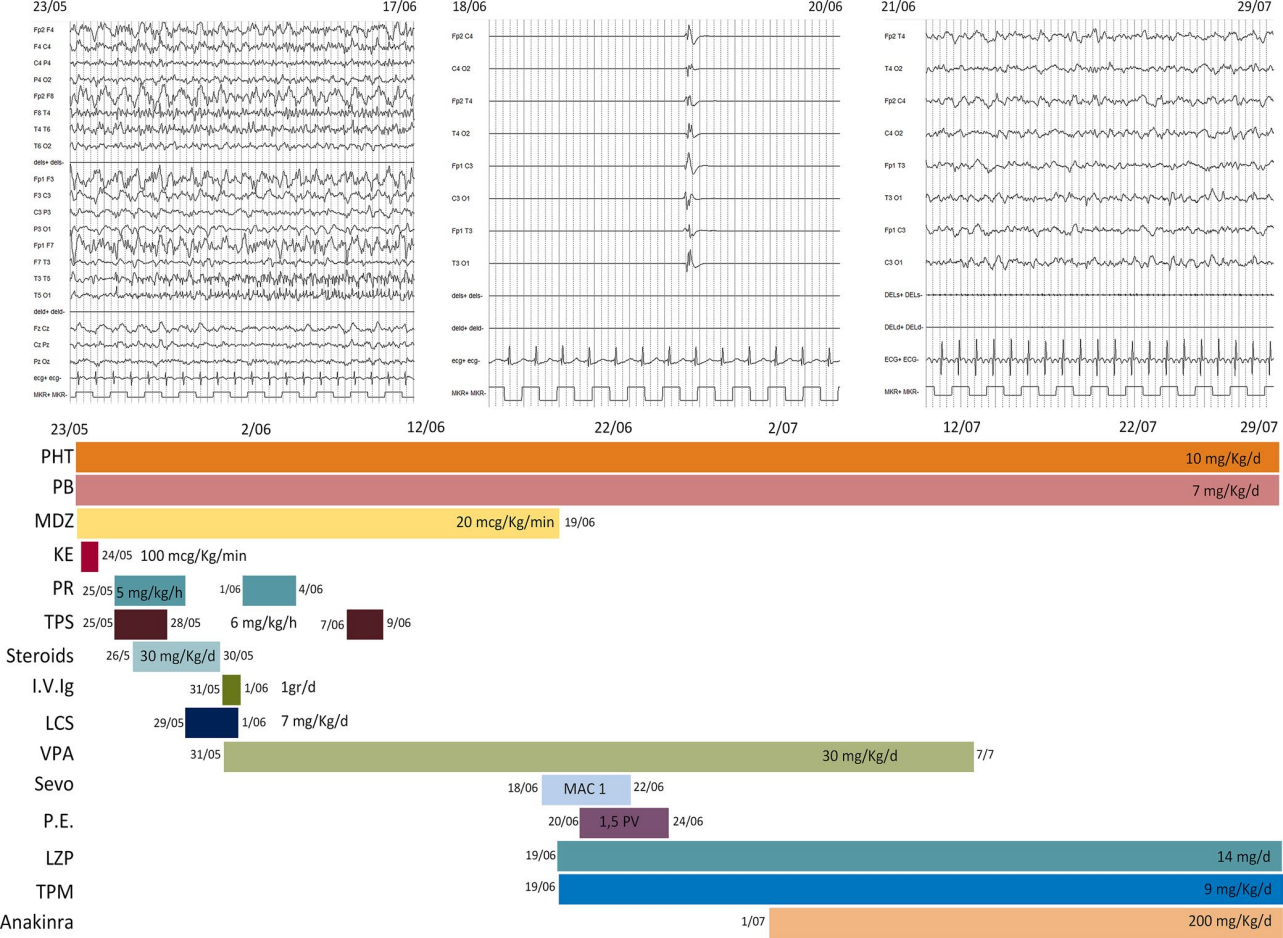
EEG features (figure above) and treatments (figure below) during the 68 days in ICU from May 25 to July 29. EEG features, ictal EEG pattern (to the left), suppression‐burst pattern (at the center) and interictal EEG activity after status epilepticus resolution (to the right). Treatments: IV Ig, intravenous immunoglobulin; KE, ketamine; LCS, lacosamide; LZP, lorazepam; MDZ, midazolam; PB, phenobarbital; PE, plasma exchange; PHT, phenytoin; PR, propofol; Sevo, sevoflurane; TPM, topiramate; TPS, thiopental; VPA, valproate

The patient was therefore enrolled in a national multicenter randomized sequential trial approved by the Italian Medicines Agency (EudraCT number 2013‐004396‐12; ClinicalTrial.gov identification number: NCT02431663) and randomized to ketamine (KE) (study arm).^13^ As per protocol, KE was started and the dose was rapidly increased up to 100 mcg/kg/min. Only transitory EEG suppression‐burst (SB) patterns were recorded during the 3 mg/kg KE bolus administration at each infusion velocity increment. Due to KE inefficacy, midazolam (MDZ) up to 12 mcg/kg/min, propofol (PR) up to 4 mg/kg/h, and thiopental (TPS) up to 5 mg/kg/h (control arm) were also administered as per protocol. A transitory reduction of seizure frequency without SB pattern appearance was obtained only with high doses of MDZ, and not with PR and TPS. Several anti‐seizure medications (intravenous lacosamide, levetiracetam and valproic acid, and topiramate and carbamazepine per os) were administered together with ketogenic diet with no substantial changes in seizure frequency and EEG features (Figure 1). Based on the seizure type and frequency, ranging from 20 to 35 per hour, EEG and clinical features, a diagnosis of FIRES was made. Status epilepticus remained unchanged despite 5 days of intravenous corticosteroids (1 g/day) and 2 days of intravenous immunoglobulins (2 g/day). Because of the refractoriness of SE, we opted for re‐administered conventional anesthetics (MDZ, PR, and TPS) and MDZ at a dosage up to 20 mcg/kg/min, thus obtaining only a transitory reduction of seizure frequency. After 29 days of prolonged SRSE, having received written informed consent by the parents, 3.5% sevoflurane (MAC 1) through a closed circuit was started. For the first time since the beginning of the seizures, a stable EEG SB pattern and resolution of SE was achieved (Figure 1). As soon as sevoflurane inhalation was reduced (MAC 0.6), focal motor seizures reappeared. Sevoflurane was increased to the previous dosage (MAC 1) in association with five‐day plasma exchange therapy. Combined therapy with sevoflurane and plasma exchange (1.5 PV) was then efficacious to reach a stable EEG SB pattern and SRSE control. No major sevoflurane‐related adverse events, such as hypotension requiring vasopressors, were observed with the exception of a transitory mild metabolic acidosis. Only a few (2 to 4 per day) minor focal motor seizures re‐occurred after the withdrawal of sevoflurane and plasma exchange. Seven days after sevoflurane and plasma exchange had been discontinued, anakinra at the dosage of 100 mg b.i.d. was started in association with PHT, PB, topiramate and lorazepam (Figure 1). Due to the poor mental status of the patient and in order to begin ventilator weaning, a tracheostomy was necessary. The patient was transferred to the Neurology Department following 68 days of intensive care. After another 12 days, he was discharged from the hospital and sent home with 2 to 4 focal motor seizures a week and in a minimally conscious state. The first brain MRI report, performed at one week from seizure onset, was normal. At one month from seizure onset, mild cerebral atrophy and hippocampal sclerosis was observed; after six months, MRI showed moderate to severe cortical‐subcortical atrophy.

## 3 DISCUSSION

In our case, inhaled anesthesia with sevoflurane combined with plasma exchange was effective in controlling a prolonged SRSE with no adverse events. An EEG SB pattern was only achieved with sevoflurane, and SRSE resolution was obtained when plasma exchange therapy was added, thus confirming the efficacy of immunotherapy in FIRES. The low blood solubility and high lipid solubility of sevoflurane are responsible for its rapid distribution in the cerebral tissue and its scarce elimination through plasma exchange, unlike the most commonly used intravenous anesthetics.^12^ These pharmacokinetic features make sevoflurane particularly interesting in treating SE in which plasma exchange is indicated.

Isoflurane is the only volatile anesthetic recommended in the treatment of RCSE.^11^ It is preferred to desflurane, xenon, and sevoflurane because it has a higher rate of tolerability and hypothetical lower toxicity.^11^ In fact, electroencephalogram abnormalities and epileptiform patterns have been observed in children undergoing sevoflurane mask anesthesia induction, both in healthy children and in children with epilepsy.^14^ Some studies showed that lower sevoflurane concentration during anesthesia induction and shorter induction time could reduce the occurrence of epileptic discharge.^15^ Kreuzer et al showed that anesthesia induction with 6% sevoflurane in children was accompanied by reduced epileptiform activity when compared to the administration of 8% sevoflurane.^15^ Benzodiazepine premedication has been considered responsible for the absence of epileptiform EEG changes during sevoflurane anesthesia.^16^

Recent experimental studies suggest that volatile anesthetics have a neuroprotective effect when the brain is undergoing ischemia, yet they display neurotoxicity under normal brain conditions when administered during early or late development stages.^17^

We report administering sevoflurane for an extremely severe epileptic episode of prolonged SRSE. It was administered along with MDZ and lorazepam. Both the ischemic condition together with the paired administration of benzodiazepines may have contributed to the reduction of its toxicity and may have enhanced its efficacy.^18^

In our case, anakinra was efficacious in maintaining seizure control after sevoflurane withdrawal, thus supporting its efficacy in FIRES.^10^

The brain MRI revealed a diffuse cortical‐subcortical brain atrophy, as is often reported in FIRES patients.^19^ Whether brain atrophy is due to prolonged intractable seizures or the disease entity itself is still uncertain.

We suggest that sevoflurane as bridge therapy for immunosuppressive treatment could be taken into consideration early in those children with SE in which an autoimmune‐inflammatory etiology may be reasonably hypothesized.

## CONFLICT OF INTEREST

The authors have disclosed that they do not have any potential conflicts of interest. The authors confirm that they have read the Journal's position on issues involved in ethical publication and affirm that this report is consistent with those guidelines.
